# *In Vitro* and *in Vivo* Studies of the Inhibitory Effects of Emodin Isolated from *Polygonum cuspidatum* on Coxsakievirus B_4_

**DOI:** 10.3390/molecules181011842

**Published:** 2013-09-25

**Authors:** Zhao Liu, Fei Wei, Liang-Jun Chen, Hai-Rong Xiong, Yuan-Yuan Liu, Fan Luo, Wei Hou, Hong Xiao, Zhan-Qiu Yang

**Affiliations:** 1 State Key Laboratory of Virology, Institute of Medical Virology, School of Medicine, Wuhan University, Wuhan 430071, China; E-Mails: liuzhao@mail.scuec.edu.cn (Z.L.); simon1985511@gmail.com (F.W.); chenliangjun1122@163.com (L.-J.C.); xionghairong@gmail.com (H.-R.X.); liuyuanyuan@126.com (Y.-Y.L.); fanluo2011@whu.edu.cn (F.L.); houwei@whu.edu.cn (W.H.); xh@163.com (H.X.); 2 College of Pharmacy, South-Central University for Nationalities, Wuhan 430074, China; 3 School of Basic Medicine, Hubei University of Chinese Medicine, Wuhan 430065, China

**Keywords:** emodin, *Polygonum cuspidatum*, inhibitory effect, Coxsakievirus B_4_

## Abstract

The lack of effective therapeutics for Coxsackievirus B_4_ (CVB_4_) infection underscores the importance of finding novel antiviral compounds. Emodin (1,3,8-trihydroxy-6-methylanthraquinone) is one of the natural anthraquinone derivatives obtained from the root and rhizome of *Polygonum cuspidatum*. In the present study, the possibility of using emodin as a potential antiviral to treat CVB_4_ infection was explored *in vitro* and in mice. Emodin reduced CVB_4_ entry and replication on Hep-2 cells in a concentration- and time-dependent manner, with a 50% effective concentration (EC_50_) of 12.06 μM and selectivity index (SI) of 5.08, respectively. The inhibitory effect of emodin for CVB_4_ entry and replication was further confirmed by a quantitative real time PCR (qPCR) assay. The results further showed that the mice orally treated with different dosages of emodin displayed a dose dependent increase of survival rate, body weight and prolonged mean time of death (MTD), accompanied by significantly decreased myocardial virus titers and pathologic scores/lesions. Moreover, emodin could inhibit CVB_4_-induced apoptosis *in vitro* and *in vivo*. Our results indicated that emodin could be used as potential antiviral in the post-exposure prophylaxis for CVB_4_ infection.

## 1. Introduction

Coxsackievirus B_4_ (CVB_4_) belongs to the genus *Enterovirus*, family *Picornaviridae*, and is one of six serotypes of the coxsackievirus B group. CVB_4_ can cause a broad range of diseases, such as myocarditis, pancreatitis, hepatitis, aseptic meningitis, meningoencephalitis, gastroenteritis, necrotizing enterocolitis, pneumonia and even death in neonates [1]. Moreover, clinical symptoms such as myopericarditis and pleurodynia (Bornholm disease) are still distinct and are associated only with CVB_4_ infections [2]. Until now, there are no enterovirus-specific vaccines or therapeutic agents available for clinical usage of CVB_4_ infection [3]. Current clinical therapeutic method and supportive treatment to reverse inflammation and alleviate the symptoms in many CVB_4_-infected patients has been still largely disappointing [4]. Although a great number of *in vitro* picornavirus replication inhibitors have been described, few of them have shown effectiveness *in vivo* [5], and none has been approved for clinical use to date. Ribavirin may act as a lethal mutagen via incorporation into the viral RNA genome. *In vitro* activity of ribavirin against enteroviruses has been demonstrated by several groups [6], and treatment of murine CVB_3_ myocarditis led to significantly decreased myocardial virus titer, inflammation, necrosis and myocardial calcification [7]. One side effect of ribavirin, however, is drug resistance [8], hemolytic anemia [9], and it is licensed only for the treatment of respiratory syncytial virus and hepatitis C virus infections [10]. Therefore, there is a need for an antiviral therapy which is effective for treating CVB_4_ infection.

Emodin (1,3,8-trihydroxy-6-methylanthraquinone) is an active component in the root and rhizome of *Polygonum cuspidatum*, which has been used in Traditional Chinese Medicine (TCM) for treating diseases like liver cirrhosis, diabetic nephropathy, atherosclerosis and tumours [11]. A number of pharmacological properties including anti-microbial, anti-inflammatory, antiviral, anticancer, immunosuppressive, and chemopreventive effects have been suggested [12,13]. Moreover, an increasing number of studies, including those from our laboratory, have extended the antiviral activity of emodin to many RNA and DNA viruses, enveloped and non-enveloped viruses, and pH-dependent and independent viruses, such as herpes simplex virus [14], hepatitis B virus [15], severe acute respiratory syndrome (SARS) coronavirus [16], *etc.* These findings are crucial for the understanding of the pharmacological properties of emodin, since despite extensive studies of its antiviral effects, the antiviral activity of emodin against CVB_4_ virus infections is still incompletely understood.

In this study, we systematically investigated the antiviral activity of emodin against CVB_4_* in vitro* and *in vivo*. We demonstrated that emodin could inhibit the entry and replication of CVB_4_ in a concentration- and time-dependent manner. Emodin could also inhibit apoptosis induced by CVB_4_ infection. The *in vivo* study further showed that oral administration of emodin could significantly mitigate myocardial virus titers and pathologic lesions induced by CVB_4_ infection. These findings suggested that emodin may represent as a potential therapeutically effective agent for CVB_4_ infection.

## 2. Results and Discussion

### 2.1. *In Vitro* Experiments

#### 2.1.1. Cytotoxicity of Emodin on HEp-2 Cells

**Figure 1:**
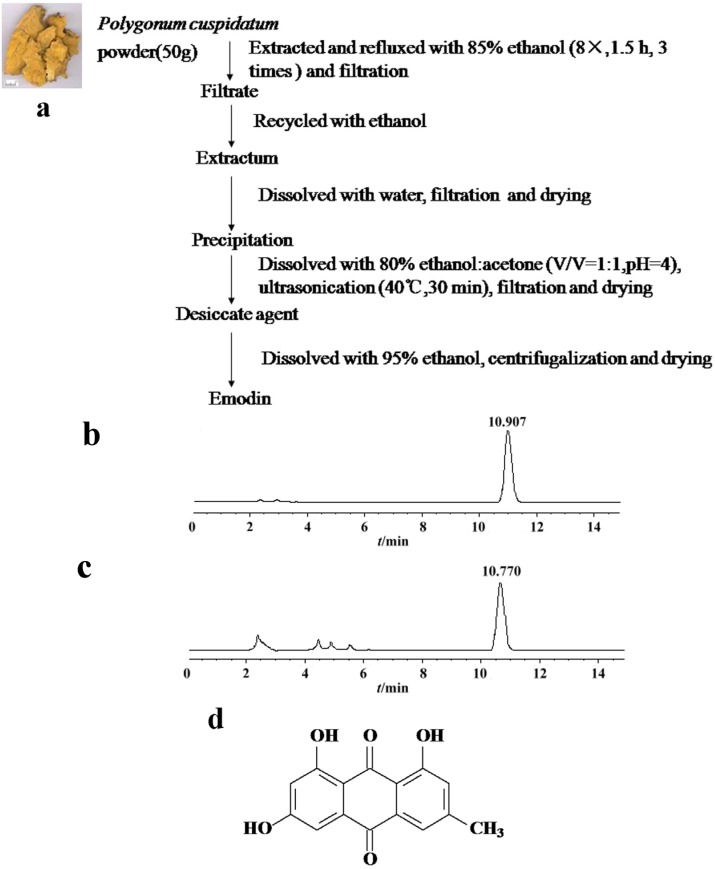
Isolation and identification of emodin (**a**) Extraction of emodin from *Polygonum cuspidatum* (**b**) HPLC analysis for emodin standard reference (**c**) HPLC analysis for emodin sample isolated from *Polygonum cuspidatum* (**d**) Chemical structure of emodin.

The extraction process and High Performance Liquid Chromatography (HPLC) analysis for emodin are shown in Figure 1a–c. The separation of emodin was carried out on an Acclaim 120-C18 column (4.6 mm × 250 mm, 5 μm) with a mobile phase of methanol-water-0.1 phosphoric acid (85:15:0.05) under 30 °C. The flow rate was set at 1.0 mL/min and the detection wavelength was 289 nm. The calibration curves of emodin showed good linearity over the 0.0075~0.0214 mg/mL range (*y* = 3197.9930*x* + 43.4724, *r* = 0.9981). The emodin extracted from 50 g *Polygonum cuspidatum* is about 560.50 ± 7.08 mg, or (11.21 ± 0.14)% in terms of dried starting materials. The purity of emodin was (70.69 ± 0.32)% in three independent experiments. We then tested the cytotoxicity of emodin on HEp-2 cells by the 3-(4,5-dimethylthiazol-2-yl)-2,5-diphenyltetrazolium bromide (MTT) assay. As shown in Table 1, the toxicity of emodin on HEp-2 cells was (61.35 ± 4.44) μM, and the highest non-toxic concentration was around 46.26 μM.

**Table 1:** Cytotoxicity and selectivity index of emodin on HEp-2 Cells.

Compound	CC_50_ (μM)	EC_50_ (μM)		Selectivity index	
	MTT	MTT	PRA	MTT	PRA
Emodin	61.35 ± 4.40	14.10 ± 0.74	12.06 ± 1.85	4.35 ± 0.6	5.09 ± 0.3
Ribavirin	2645.14 ± 54.05	629.99 ± 1.64	460.91 ± 1.23	4.20 ± 0.7	5.74 ± 0.1

Cytotoxicity was determined by MTT assay. Antiviral activity was determined by MTT and PRA. Selectivity index = CC_50_ (50% cytotoxic concentration)/EC_50_ (50% effective concentration). Emodin: 1 μM = 270.24 ng/mL; Ribavirin: 1 μM = 244.21 ng/mL.

#### 2.1.2. Inhibitory Effect of Emodin on CVB_4_ Infection

**Figure 2:**
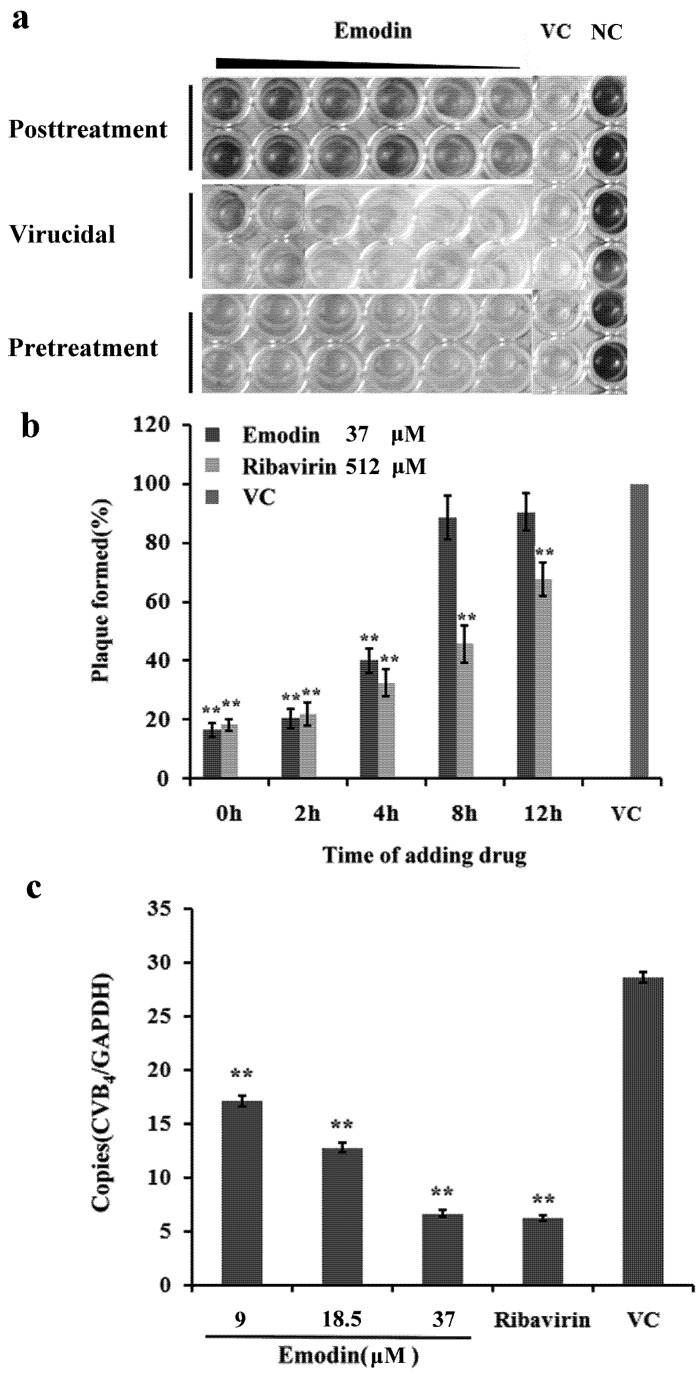
Antiviral activity of emodin against CVB_4_ infection. (**a**) MTT assay was performed to determine antiviral effect of emodin on HEp-2 cells. NC and VC indicate normal control and viral control. (**b**) Time-of-addition assay. HEp-2 cells were seeded into 12-well culture plates and were infected with CVB_4_ for 1 h at 4 °C to allow virus binding but not virus entry, the infected cells were treated or mock treated with emodin (37 μM) or ribavirin (512 μM) or 2% test medium at the indicated times intervals of 0, 2, 4, 8, and 12 h postinfection. Virus titers were determined by plaque assay. The data are expressed as the mean ± SD of three independent experiments. ** *p* < 0.01 versus virus control. (**c**) Dose-response columns for emodin treatment in HEp-2 cells after drug incubation as determined by qPCR assay. The CVB_4_ RNA levels were normalized to that of the housekeeping gene GAPDH. Each data point represents the mean ± standard deviation from three independent experiments. ** *p* < 0.01 versus virus control.

Several reports have shown that emodin is active against multiple viral infections. We initially tested the ability of emodin to inhibit CVB_4_ entry and replication in HEp-2 cells. It has been reported that the CC_50_ of emodin on HepG2.2.15 cells was 150.46 μM. The IC_50_ against HBV was 77.71 μM [17]. Cells were first infected with CVB_4_ and then treated with different concentrations of emodin. The virus-induced cytopathic effect (CPE) was delayed dramatically by emodin in a concentration-dependent manner (Figure 2a). As shown in Table 1, the EC_50_ of emodin determined by the MTT and plaque reduction assay (PRA) were 14.10 ± 0.74 μM and 12.06 ± 1.85 μM, respectively. The selectivity indexes (SI) of emodin evaluated by MTT and PRA were 4.35 ± 0.6 and 5.09 ± 0.3, respectively. The inhibitory effect of emodin against CVB_4_ infection was equivalent to that of the conventional antiviral ribavirin. There was no significant difference of SI between emodin-treated group and ribavirin-treated group (*p* > 0.05). To determine whether the inhibition of the CVB_4_ entry and replication by emodin was also time dependent, the compound was added at indicated times (0, 2, 4, 8, 12 h postinfection). The maximum inhibitory rate could be observed when emodin was added at 0–4 h postinfection when placed at 37 °C after inoculation (Figure 2b). Published research by several other investigators has indicated that the mechanism of antiviral activity of emodin derived from other sources relies on direct inactivation of infectious viral particles [18,19]. To investigate if emodin had a direct inactivation effect on CVB_4_ particles, varying concentrations of emodin were incubated with CVB_4_ for indicated times prior to inoculation on cells. However, no virucidal effect could be observed in our study, with the maximum inhibitory rate of emodin in virucidal assay being only about (8.26 ± 0.3)%. No inhibitory activity was observed when cells were pre-incubated with various concentrations of emodin, since the inhibitory rate was only (7.35 ± 0.2)%~(9.56 ± 0.5)% at each dosage. There was no significant difference between emodin-treated groups and the virus control group in the direct inactivation and pre-incubation assay (*p* > 0.05). Quantitative real time PCR (qPCR) was then used to evaluate CVB_4_ RNA levels. Figure 2c summarizes the number of copies obtained from different treatments after normalizing to glyceraldehyde-3-phosphate dehydrogenase (GAPDH). It was observed that CVB_4_ copies in the 37, 18.5, 9.0 μM emodin group expressed 0.77, 0.55 and 0.40-fold lower than the virus control group (*p* < 0.01), a result that corroborates the dose dependent inhibitory effect of emodin against CVB_4_ infection.

#### 2.1.3. Emodin Mitigates CVB_4_-Induced Apoptosis *in Vitro*

**Figure 3:**
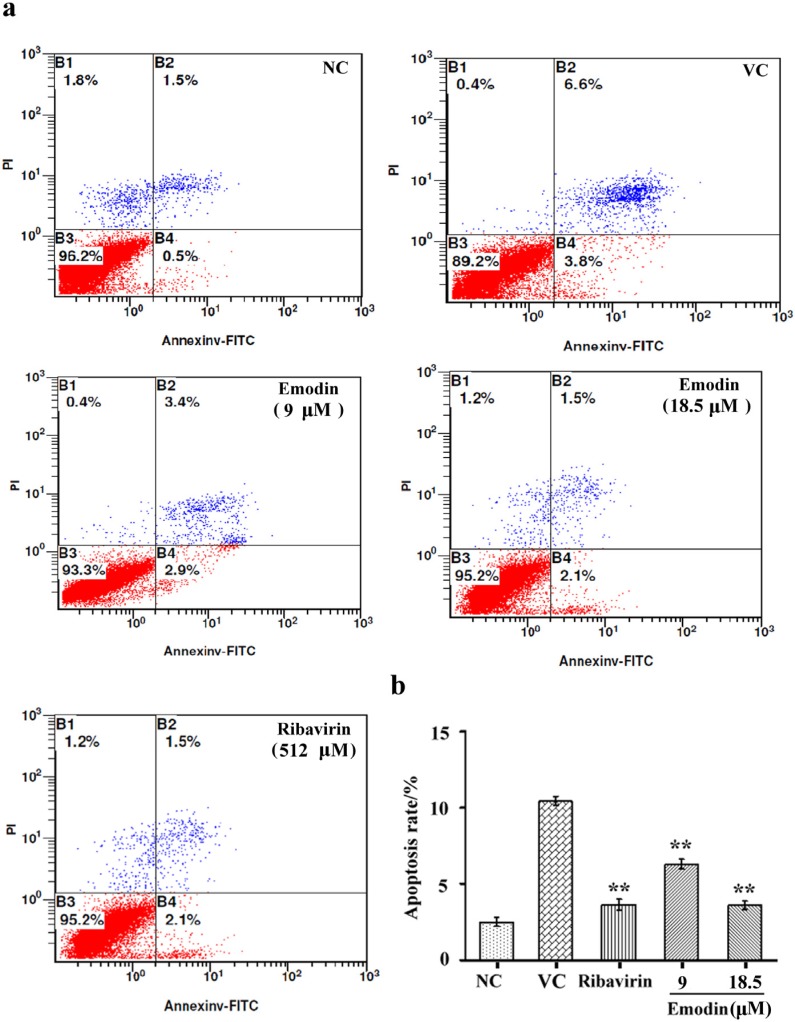
Effects of emodin on apoptosis in HEp-2 cells induced by CVB_4_ infection. (**a**) Hep-2 cells were infected with CVB_4_ and incubated with emodin (18.5, 9 μM) or ribavirin (512 μM) for 48 h, stained with Annexin-V and PI, and analyzed by flow cytometry. (**b**) Column bar graph of apoptosis. ** *p* < 0.01 *versus* virus control.

Previous data showed that CVB_4_ could induce apoptosis in primary neurons. To assess the hypothesis that emodin may attenuate the ongoing cell death or apoptosis in HEp-2 cells after CVB_4_ infection, HEp-2 cells infected with CVB_4_ were incubated with 18.5, 9 μM emodin or 512 μM ribavirin and harvested to evaluate apoptotic changes. The results are summarized in Figure 3. The number of cells with Annexin-V binding was almost equal, but the apoptosis rate was significantly increased in cells infected with CVB_4_ virus at 48 h postinfection. The apoptosis rate in cells treated with emodin and ribavirin were significantly lower than the infected controls (*p* < 0.01), suggesting that emodin can inhibit apoptosis induced by CVB_4_. There were no differences between the 18.5 μM emodin and 512 μM ribavirin (*p* > 0.05) treated groups.

### 2.2. *in Vivo* Experiments

#### 2.2.1. Emodin Increases the Survival Rate of CVB_4_ Infected Mice

The median lethal dose (LD_50_) of emodin was determined prior to virus inoculation. Death rate for oral gavage of emodin at the dosages of 2.00, 1.00, 0.50, 0.25, 0.12 g/kg/d for 10 d were 100%, 83%, 33%, 8% and 0%, respectively. LD_50_ of emodin was calculated as 0.58 g/kg/d. The pharmacokinetics of emodin and ribavirin were determined by HPLC analysis, respectively. The pharmacokinetic model of emodin and ribavirin fits well with the two-compartment model. Plasma levels of emodin (30 mg/kg/d) rise to the maximum 10 min after administration, with C_max_ at 21.6 ± 2.23 μM. The half life is 11.5 h. Plasma concentration of ribavirin rises to the maximum 12 min after administration, with C_max_ at 5.357 ± 0.312 μM. The half life is 12 h.

Clinical signs of murine CVB_4_ infection that generally appeared 3 d post-infection were observed in some mice, especially in the virus control. Changes in behavior, such as transient states of hyperactivity and weight loss, accompanied by hunched posture and progressively diminishing vitality were observed. Compared with the placebo-treated animals, emodin-treated animals died later and the appearance of clinical signs was delayed in a dose-dependent manner.

**Figure 4:**
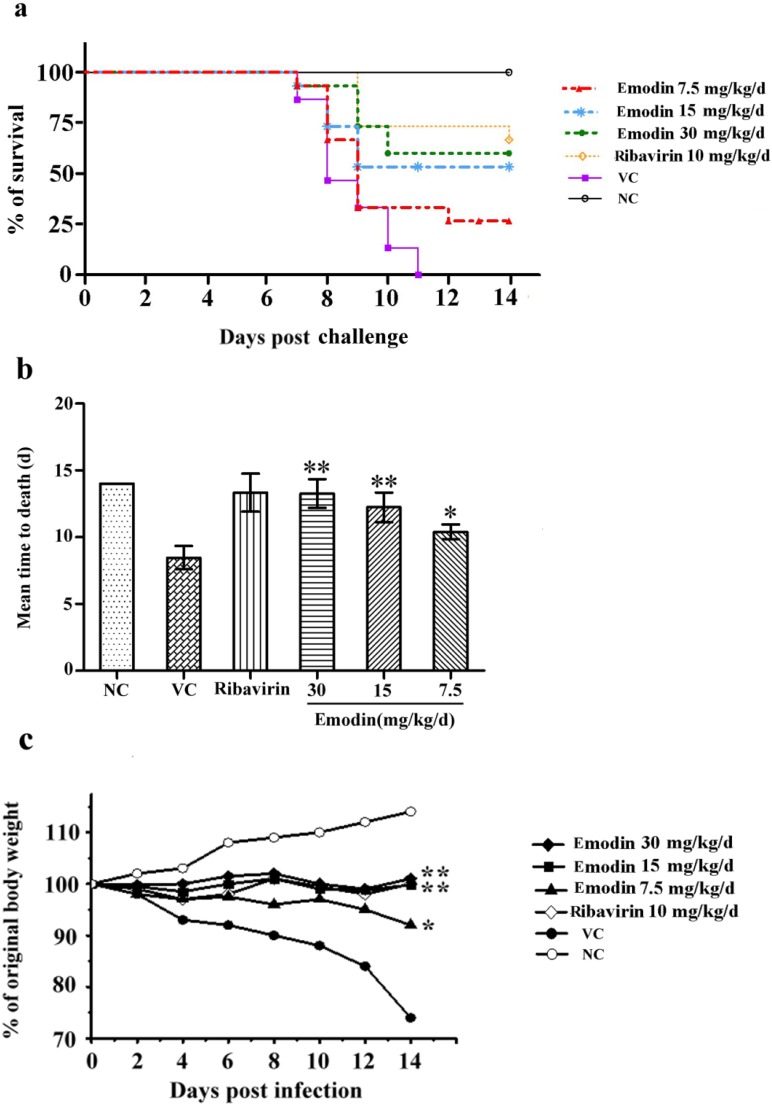
Mortality following CVB_4_ infection and emodin treatment in mice. Mice challenged with 10 LD_50_ of CVB_4_ were orally administered with 30 mg/kg/d, 15 mg/kg/d, and 7.5 mg/kg/d emodin, respectively. 0.9% saline was used in viral control and normal control group. The survival rate (**a**), mean time to death (**b**), and body weight (**c**) of each group were determined. Statistical significance is determined by Kaplan-Meier Log Rank Test, ** *p* < 0.01, * *p* < 0.05 *vs.* infected control group.

As shown in Figure 4a, the survival rate of the 30 mg/kg/d (9/15, 60.0%, *p* < 0.01), 15 mg/kg/d (8/15, 55.33%, *p* < 0.01) and 7.5 mg/kg/d (4/15, 26.67%, *p* < 0.01) groups increased dramatically compared to the virus control group. The 30 mg/kg/d and 15 mg/kg/d emodin-treated groups showed no significant difference with the ribavirin group (10/15, 67. 67%, *p* > 0.05) and the 30 mg/kg/d group showed a better survival rate than 7.5 mg/kg/d group (*p* < 0.05). In Figure 4b, compared to virus group (8.47 ± 0.87 d), MTD was prolonged in treatment of emodin at the dosage of 30 mg/kg/d (13.27 ± 1.08 d, *p* < 0.01), 15 mg/kg/d group (12.23 ± 1.10 d, *p* < 0.01) and 7.5 mg/kg/d group (10.4 ± 0.56 d, *p* < 0.05). MTD showed no differences among 30 mg/kg/d, 15 mg/kg/d emodin-treated groups and ribavirin control (13.33 ± 1.43, *p* > 0.05).

The body weights of the animals were also measured for 14 consecutive days (Figure 4c). Mice infected with virus without treatment exhibited an obvious weight loss compared to the uninfected mice (*p* < 0.01). However, mice treated with emodin maintained relatively steady weights and showed less weight loss throughout the infection. The groups treated with 30 mg/kg/d and 15 mg/kg/d emodin showed no differences with the ribavirin-treated control after 8 d (*p* > 0.05).

#### 2.2.2. Emodin Alleviates the Myocardial Lesions Induced by CVB_4_ Infection

**Figure 5:**
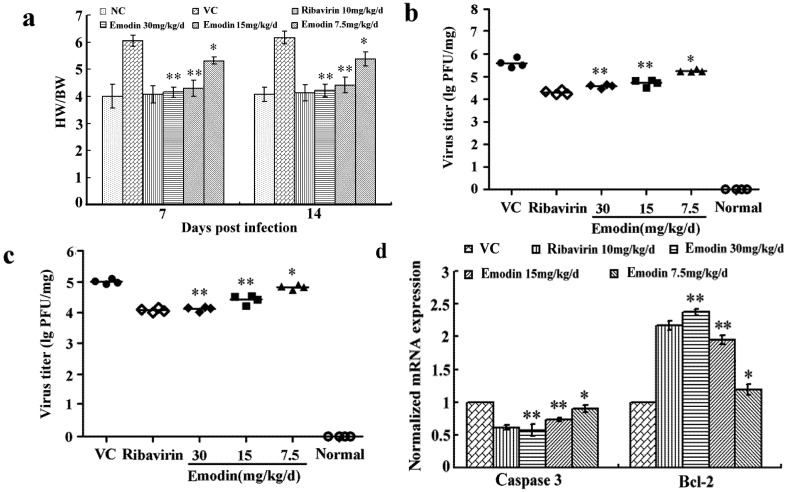
Emodin treatment alleviates myocardial inflammation and reduced apoptosis-related genes expression in CVB_4_ infected mice. (**a**) Mice were sacrificed at indicated times. Body weight (BW/g) and heart weight (HW/mg) of each group was measured to calculate the heart to body weight ratio (HW/BW) for each group. (**b**, **c**) Heart virus titers were determined by plaque assay on the 7th day (**b**) and 14th day (**c**) postinfection. (**d**) The mRNA expression of caspase-3 and bcl-2 in treated and control groups were determined by qPCR assay. ** *p* < 0.01, * *p* < 0.05 *vs.* infected control group.

**Figure 6:**
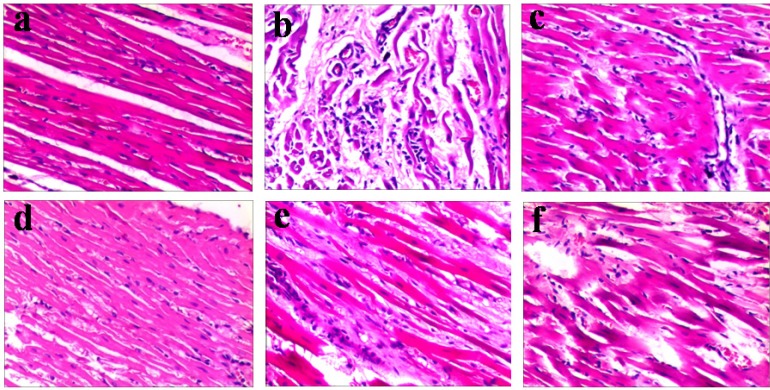
Emodin treatment ameliorates CVB_4_ induced heart lesions**.** Photomicrographs of H&E-stained paraffin sections generated from (**a**) normal control (**b**) viral control (**c**) ribavirin (10 mg/kg/d) (**d**) emodin (30 mg/kg/d) (**e**) emodin (15 mg/kg/d) (**f**) emodin (7.5 mg/kg/d). Sections from viral control displayed mononuclear cell inflammation and the appearance of multiple foci in necrotic cardiomyocytes. In groups treated with emodin or ribavirin, the lesions of the myocardium were relieved and the area of necrosis and inflammatory infiltrates was significantly decreased compared with non-treated, infected animals.

The heart weight/body weight (HW/BW) ratio in the infected control group on days 7 and 14 were significantly increased compared with those in the normal control group. However, the ratio decreased dramatically in mice treated with emodin at all dosages (*p* < 0.01 at 30 and 15 mg/kg/d, *p* < 0.05 at 7.5 mg/kg/d, Figure 5a). The changes of the virus titers in heart of the CVB_4_ infected mice from different groups at day 7 and 14 are shown in Figures 5b,c. Oral gavage of emodin at the dosages of 30 and 15 mg/kg/d significantly reduced the virus titers in the heart homogenates compared to the virus control group (*p* < 0.05). We further examined the myocardial lesions and evaluated the pathologic score in BALB/c mice treated with different dosage of emodin. Compared with the infected control group, the damage of myocardium was relieved and scores of necrosis and infiltration were decreased significantly with emodin treatment at all dosages 14 d after infection (Table 2 and Figure 6).

**Table 2:** Effects of emodin on pathologic score in BALB/c mice infected by CVB_4_ at 7 and 14 d postinfection.

Group	Pathologic score (means ± S.D.)			
	7 day		14 day	
	Inflammatory infiltration	Necrosis	Inflammatory Infiltration	Necrosis
NC	0.93 ± 0.67	0.90 ± 0.58	0.85 ± 0.64	0.80 ± 0.48
VC	3.04 ± 0.29	3.01 ± 0.45	3.18 ± 0.44	2.94 ± 0.39
Ribavirin 10 mg/kg/d	1.19 ± 0.17 **	1.13 ± 0.25 **	1.12 ± 0.46 **	1.05 ± 0.25 **
Emodin 30 mg/kg/d	1.15 ± 0.24 **	1.07 ± 0.31 **	1.07 ± 0.33 **	0.99 ± 0.25 **
Emodin 15 mg/kg/d	1.49 ± 0.22 **	1.39 ± 0.39 **	1.41 ± 0.20 **	1.33 ± 0.30 **
Emodin 7.5 mg/kg/d	2.08 ± 0.12 *	2.01 ± 0.16 *	2.00 ± 0.22	1.98 ± 0.23 *

** *p* < 0.01, * *p* < 0.05 *vs.* infected control group.

#### 2.2.3. Emodin Can Alter Transcription and Protein Levels of Apoptosis-Related Genes *in Vivo*

**Figure 7:**
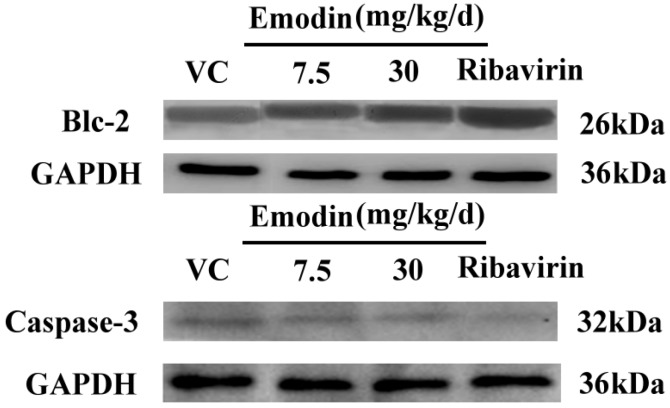
Detection of caspase-3 and Bcl-2 expression in emodin-treated (30, 7.5 mg/kg/d), ribavirin (10 mg/kg/d) and virus control groups by western blot analysis. GAPDH was examined to normalize any differences in loading.

We then assessed the expression of two apoptosis-related genes, caspase-3 and bcl-2 at both mRNA and protein levels, in order to investigate the effect of emodin on apoptosis-related genes *in vivo* at 14 d postinfection*.* Treated groups were compared against those of controls using the relative quantification (2^−^^△△^T) method. The mRNA expression levels of caspase-3 in 30, 15, 7.5 mg/kg/d emodin treated groups were 0.57, 0.73, 0.9-fold and the bcl-2 levels were 2.37, 1.95, 1.18-fold, respectively, with respect to the expression in virus control (Figure 5d). Therefore, emodin treatment could reduce the amount of caspase-3 mRNA levels, while it dramatically increased the bcl-2 mRNA levels compared to CVB_4_-infected control (*p* < 0.01 at 30 and 15 mg/kg/d, *p* < 0.05 at 7.5 mg/kg/d). The group treated with 30 mg/kg/d emodin showed no difference with the ribavirin-treated group (*p* > 0.05, 0.61-fold caspase-3 and 2.17-fold bcl-2 of virus control). The heart tissues were also analyzed to determine the expression of apoptosis-related proteins by western blot. As shown in Figure 7b, treatment with emodin (30 mg/kg/d) notably decreased the expression levels of caspase-3 while it up-regulated the expression of anti-apoptosis protein bcl-2 compared with nontreated infected groups. These results suggested that emodin could alter the transcription levels of apoptosis-related genes *in vivo*.

## 3. Experimental

### 3.1. Extraction and Isolation of Emodin

*Polygonum cuspidatum* was purchased from Hubei Medicinal Material Company (Wuhan, China). The identification and authenticity of the plant was confirmed by a professional medicinal plant expert, Prof. Keli Chen, Department of Plant Chemistry, Hubei University of Traditional Medicine (Hubei, China). The extraction under reflux scheme is shown in Figure 1a. The quantity of emodin was determined by HPLC (Dionex Ultimate 3000, Sunnyvale, CA, USA) analysis. The emodin was identified by NMR (AM-300, Bruker, Billerica, MA, USA) as follows: ^1^H-NMR (300 MHz, DMSO-d_6_) δ: 7.08 (1H, s, H-2), 7.50 (1H, s, H-4), 7.22 (1H, d, *J* = 1.0 Hz, H-5), 6.60 (1H, d, *J* = 1.0 Hz, H-7), 2.35 (3H, s, CH_3_), 12.09, 12.16 (1H each, s, OH); ^13^C-NMR (75 MHz, DMSO-d_6_) δ:164.84 (C-1), 120.05 (C-2), 147.60 (C-3), 123.59 (C-4), 109.32 (C-5), 161.72 (C-6), 108.30 (C-7), 166.38(C-8), 189.85 (C-9), 181.50 (C-10), 133.42 (C-4a), 108.71 (C-8a), 113.15 (C-9a), 134.76 (C-10a), 22.17 (CH_3_).

### 3.2. Experiments *in Vitro*

#### 3.2.1. Cells and Virus Stocks

HEp-2 (human laryngeal carcinoma) cells were routinely grown in DMEM (Dulbecco’s modified Eagle’s medium) supplemented with 10% fetal calf serum (Gibco, Langley, OK, USA), penicillin (100 IU/mL), streptomycin (100 μg/mL) and 0.1% L-glutamine. Coxsackie virus B_4_ strain was maintained in our laboratory and propagated in HEp-2 cells. The virus titer was estimated from cytopathogenicity of cells induced by viral infection and expressed as 50% tissue culture infectious doses (TCID_50_) [20].

#### 3.2.2. Evaluation of Cytotoxicity

The MTT cell proliferation assay was performed as described previously [21]. Briefly, cells were seeded in 96-well plates and cultured at 37 °C in an atmosphere of 5% CO_2_ incubator for 24 h until 80%~90% confluent monolayer were formed. After removal of the growth medium, two-fold serial dilutions of emodin and ribavirin (Tianjing Pharmaceutical Co. Ltd., Tianjing, China) were added and incubated for 72 h. Then the culture medium was removed and the 50 μL thiazolyl blue tetrazolium bromide solution (Sigma-Aldrich, St. Louis, MO, USA) was added and incubated for 2~4 h until the purple precipitate was fully visible. The formazan was dissolved in dimethyl sulfoxide (DMSO), and then quantitated in a conventional microplate reader at 490 nm according to the manufacturer’s protocol. The toxic dose for 50% cell death (CC_50_) of emodin were calculated from dose-response curves by optical density (OD_of treated cells_/OD_of untreated cells_) × 100. All assays were performed in triplicate.

#### 3.2.3. Drug Treatment after Infection

HEp-2 cells grown in 96-wells cell culture plate were challenged with 100 TCID_50_ CVB_4_ for 1 h. After thoroughly washing unbound virus, serial two-fold dilutions of emodin (10, 5, 2.5, 1.25, 0.6, 0.3 μg/mL) were added to each well. The development of CPE was monitored daily by light microscopy until the virus-infected, untreated cells showed CPE up to 80%. At this time point, the antiviral activity of each concentration was measured by an MTT assay. The percent of inhibition was calculated by the following formula:

Percent of inhibition (%) = (OD_compound treated group_ − OD_virus control_)/(OD_normal control_ − OD_virus control_) × 100%.

The EC_50_ was calculated by regression analysis of the dose-inhibition relationship using SPSS 13.0. The selectivity index (SI) was evaluated with the following formula: SI = CC_50_/EC_50_.

#### 3.2.4. Virucidal Assay

The direct effect of emodin on CVB_4_ infectivity was evaluated according to the protocols described by Xiong [22] *et al.* with slight modiﬁcations. Briefly, viral suspensions containing 100 TCID_50_ of CVB_4_ were either incubated with an equal volume of medium containing different concentrations of emodin (10, 5, 2.5, 1.25, 0.6, 0.3 μg/mL) or with drug-free vehicle at 4 °C for 2–6 h, respectively. After 1 h adsorption, the mixed suspensions were removed. The cell monolayers were rinsed carefully with phosphate buffered solution (PBS) and maintained in test medium at 37 °C in a humidified atmosphere for 72 h. The virucidal effect was determined by an MTT assay following the procedures described above.

#### 3.2.5. Drug Treatment before Infection

Confluent HEp-2 monolayer cells were preincubated with fresh medium in the presence or absence of emodin (10, 5, 2.5, 1.25, 0.6, 0.3 μg/mL) for 2–6 h. The cells were then washed with PBS for twice and challenged with 100 TCID_50_ of CVB_4_ for 1 h. After virus adsorption, the cells were rinsed twice with PBS and overlaid with 2% DMEM until typical CPE was visible. The inhibition of virus was evaluated by MTT assay.

#### 3.2.6. Plaque Reduction Assay

The antiviral activity of emodin was also evaluated by a plaque reduction assay. Briefly, HEp-2 cells were seeded into 24-well culture plates and incubated until reaching at least 95% confluence. Cells were then infected with 100 TCID_50_ CVB_4_. After 1 h adsorption, the cells were washed twice with pre-warmed PBS, and overlaid with 1.2% agarose (42–45 °C) containing complete DMEM with different concentration of emodin and ribavirin. The cells were then fixed with 10% formaldehyde for 30 min and stained with 1% crystal violet solution. The plaques numbers were counted by visual examination and percentage of plaque inhibition was calculated following the protocols described elsewhere.

#### 3.2.7. Time of Addition Assay

Time of addition assay was performed as described elsewhere [23]. Briefly, HEp-2 cells were seeded into 12-well culture plates (Costar) at a density of 2 × 10^5^ cells/well for 24 h. Cell monolayers were then infected with 100 TCID_50_ CVB_4_ at 4 °C for 1 h. The unbound virus was removed from the monolayers with PBS for three times. Emodin (37 μM) or ribavirin (512 μM) was added into wells concurrently with CVB_4_ infection (0 h) or at intervals of 2, 4, 6, 8, 10 and 12 h after infection. The virus titers were determined by plaque assay as described previously.

#### 3.2.8. Quantification of CVB_4_ RNA Level

Total RNAs were isolated using TRIzol Reagent (Invitrogen, Carlsbad, CA, USA) according to manufacturer’s instructions. Random primer (Sangon, Shanghai, China) was used for reversed transcription of the total RNA by M-MLV reverse transcriptase (Promega, Beijing, China). cDNAs obtained from reverse transcription were stored at −20 °C. Primer pairs referred to reference [24] are listed as following (130 bp): CVB_4_ forward primer 5'-GTAGTCCTCCGGCCCCT-3'; reverse primer 5'-AATTGTCACCATAAGCAGCCA-3'.GAPDH (122bp) forward primer 5'-TCATTGACCTCAACTACATGGTTT-3'; reverse primer 5'-GAAGATGGTGATGGGATTTC-3'. The reaction was performed on a Bio-Rad CFX96 instrument using SYBR Green Real-time PCR Master Mix Reagent (Toyobo, Japan). Each real-time PCR reaction was performed by initial denaturation at 95 °C for 3 min, then a three-step cycle procedure (denaturation, 95 °C 10 s; annealing, 55 °C 10 s; extension, 72 °C 30 s) for 40 cycles, 95 °C 10 s, 65 °C 5 s, end. All the reactions were performed in triplicate and the results were normalized to GAPDH. The fluorescence emission of each cycle was monitored and analyzed using Bio-Raid CFX manger software.

#### 3.2.9. Detection of Apoptosis by Flow Cytometry

The apoptotic cells were assessed by flow cytometry using Annexin-V/propidium iodide (PI) apoptotic kit according to manufacturer’s protocol (Promega). In brief, Hep-2 cells infected with CVB_4_ were treated with emodin for 48 h. Floating cells were collected and centrifuged at 500 g for 10 min. Cells pellets were washed twice with ice cold PBS, resuspended in 500 µL 1 × binding buffer containing 5 µL FITC-conjugated annexin-V antibody and 8 µL of PI (5 µg/mL) for 10 min at room temperature in the dark. Apoptotic cells were determined by ﬂow cytometry and the data were analyzed using Cell Quest software (EPICS ALTRA II, Beckman, Brea, CA, USA).

### 3.3. Experiments *in Vivo*

#### 3.3.1. Experimental Design and Tissue Sampling

BALB/c mice were purchased from the Animal Research Center of Wuhan University (Certificate No. SCXK 2008-0004, Hubei, China). Mice were handled in accordance with guidelines approved by the Institutional Animal Care and Use Committee (Wuhan, China). Ninety male BALB/c mice of 4~6 weeks in age were randomly divided into six groups. Mice were infected by intraperitoneal injection with 0.2 mL saline containing 10 LD_50_ of CVB_4_. Mice were orally administered with the indicated doses (30, 15, 7.5 mg/kg/d) of emodin or 10 mg/kg/d ribavirin for 14 consecutive days, respectively. Saline (0.9%) was used in normal and viral controls. Body weight and death were recorded each day. Eight mice from each group were sacrificed on days 7 and 14 after viral inoculation. The cardiac index was expressed as the ratio of mean heart weight to mean body weight. The heart was divided into two parts, one part was homogenized in DMEM to determine virus titers by plaque assay, and the other was fixed for further histological examinations. Histopathological features of myocarditis, including necrosis and inflammation were graded by a semiquantitative score from 0 to 4 (0 = no necrosis or inflammation, 1 = 1~10 foci of necrosis or inflammation per section, 2 = 11~20 foci, 3 = 21~40 foci, 4 = over 40 foci or confluent areas of necrosis).

#### 3.3.2. Apoptosis-Related Genes Isolation and Real-Time RT-PCR

Total RNAs were extracted with Trizol reagent from heart (Invitrogen) and reverse transcripted into cDNA, according to the manufacturer’s protocol. Primers were designed by Primer Premier 5 software and listed as follows: caspase-3: 5'-TGAGGCGGTTGTAGAAGAG-3', 5'-TAATGAGAATGGGGGAAGA-3'; Bcl-2: 5'-GATTGATGGGATCGTTGCCTTA-3', 5'-CCTTGGCATGAGATGCAGGA-3'. After an initial denaturation step at 94 °C for 3 min, a three-step cycle procedure was carried out (denaturation, 95 °C, 15 s; annealing, 58 °C, 15 s; and extension,72 °C, 40 s) for 40 cycles. All reactions were performed in at least duplicate for each sample. The relative mRNA expressions were normalized to the level of GAPDH and were calculated using the formula as described elsewhere [25].

#### 3.3.3. Immunoblot Analysis

Infected cardiac muscle tissues, either untreated or with different treatment, were cut and centrifuged at 3,000 × g at 4 °C for 5 min. The remaining tissues were homogenated for 30 s in lysis buffer at 0 °C and centrifuged at 12,000 × g at 4 °C for 10 min.400 μL supernate were mixed with 5× loading buffer , boiled for 5 min, then centrifuged at 12,000 × g at 4 °C for 10 min. The resulting supernatants were loaded on a 12% SDS-polyacrylamide gel for electrophoresis and transferred to nitrocellulose membranes. Membranes were blocked with 5% dry milk solution at 4 °C overnight and washed twice with trans-buffer (without methanol) before incubated with antibodies against mouse caspase-3, rabbit anti-Bcl-2 and mouse anti-GAPDH (Beyotime Institute of Biotechnology, Co., Ltd., Nanjing, China). The membranes were washed and incubated with horseradish peroxidase-conjugated secondary antibodies in blocking solution. Protein bands were visualized with an enhanced chemiluminescence (ECL) detection system.

### 3.4. Statistical Analysis

Each set of experiments was repeated at least three times with consistent results. The data were analyzed by SPSS 13.0 software (SPSS Inc., Chicago, IL, USA). The data of *in vitro* experiments was analyzed using Student’s t-test, and that of *in vivo* experiments was analyzed using Log-rank test for survival rates, analysis of variance (ANOVA) for MTD and Kaplan-Meier method for survival analysis. A *p-*value of < 0.05 was considered statistically significant.

## 4. Conclusions

Our results demonstrated the potent antiviral activity of emodin by inhibiting CVB_4_ entry and replication, especially during the first 0–4 h postinfection*.* Our results also implied that emodin may act as a biological synthesis inhibitor against CVB_4_ infection rather than directly inactivating the viruses or blocking their absorption to the susceptible cells. The inhibitory effect of emodin on CVB_4_ replication further confirmed by qPCR assay was equivalent that of the conventional antiviral ribavirin. Ribavirin, a synthetic nucleoside, structurally related to inosine and guanosine, can induce lethal mutations when it is incorporated into viral RNA. The antimicrobial mechanisms of emodin, however, are still largely unknown. Previous studies have revealed that emodin may directly inhibit host cell casein kinase 2 (CK2), which is essential for the phosphorylation of some viral proteins [26,27]. Moreover, the antiviral capacity of emodin against enveloped viruses is due to its affinity for phospholipid membrane and weakeness for hydrophobic interactions between hydrocarbon chains in phospholipid bilayers [13,19]. Emodin can also inhibit the 3a ion channel of coronavirus SARS-CoV and HCoV-OC43 as well as virus release from HCoV-OC43 with a K*_1/2_* value of about 20 μM [16]. And emodin may exert its antiviral activity by direct inhibiting UL12 alkaline nuclease activity of HSV-1 [14,28]. These findings suggest that emodin may be a potential antiviral candidate with a broad spectrum of antiviral activities by targeting viruses or virus-related proteins. Apoptosis may play a primary role in the pathogenesis during viral infection. In our study, emodin could also mitigate CVB_4_-induced apoptosis *in vitro* and *in vivo*. The rate of apoptosis in HEp-2 cells treated with emodin (18.5, 9 μM) was significantly lower than that of virus infected controls. There were no differences between 18.5 μM emodin and 512 μM ribavirin in inhibiting CVB_4_ apoptosis. Furthermore, emodin could down-regulate the apoptosis-promoting gene caspase-3 while up-regulate the apoptosis-inhibiting gene Bcl-2 in murine heart tissues. *In vivo* orally administered emodin at 30, 15, 7.5 mg/kg/d significantly improved mice survival rate, prolonged the MTD, decreased HW/BW, virus titers and myocardial pathologic scores caused by virus infection. 30, 15 mg/kg/d emodin treatment was equivalent to 10 mg/kg/d ribavirin in alleviating the virus myocardial lesions. These properties added further appeal to emodin as a potential therapeutic agent against CVB_4_ infection and therewith associated myocarditis.
